# Effects of bevacizumab on the neovascular membrane of proliferative diabetic retinopathy: reduction of endothelial cells and expressions of VEGF and HIF-1α

**Published:** 2012-01-01

**Authors:** Xiao-Xia Han, Chang-Mei Guo, Yue Li, Yan-Nian Hui

**Affiliations:** Department of Ophthalmology, Xijing Hospital, Fourth Military Medical University, Xi’an, Shaanxi, China

## Abstract

**Purpose:**

Anti-vascular endothelial growth factor (VEGF) agents have recently been used intravitreally during the perioperative period for proliferative diabetic retinopathy (PDR). However, the mechanism of theraputic effects of the agents remains unclear. This study aimed to investigate the effects of intravitreal bevacizumab (IVB) on retinal vascular endothelial cells and expressions of VEGF and hypoxia inducible factor-1α (HIF-1α) in PDR.

**Methods:**

Twenty-four patients with PDR were enrolled and randomized to two groups. Twelve eyes of 12 patients of each group received either an intravitreal injection of 1.25 mg bevacizumab or a sham injection 6 days before vitrectomy. Neovascular membranes (NVMs) were collected during pars plana vitrectomy. The numbers of vascular endothelial cells in the NVMs were counted after staining with hematoxylin and eosin and von Willebrand. The expressions of VEGF and HIF-1α in the NVMs were detected through immunohistochemistry. Ten epiretinal membrane specimens from patients with proliferative vitreoretinopathy (PVR) without IVB treatment were set as an additional control.

**Results:**

The number of vascular endothelial cells in NVMs of the IVB pretreated group was significantly lower than that of the sham group (21.5±3.94 versus 41.33±7.44, p=0.003). The IVB pretreated group also showed significantly lower levels of VEGF and HIF-1α in NVMs than those of the sham group (*P*_HIF-1α_=0.02, *P*_VEGF_<0.001). A stepwise regression analysis showed that IVB was a significant negative predictor for the numbers of vascular endothelial cells (β=–0.89, p<0.001) and the expressions of VEGF (β=–0.85, p<0.001) and HIF-1α (β=–0.64, p=0.001) in PDR patients. Epiretinal membranes of the PVR group showed negative staining of VEGF and HIF-1α.

**Conclusions:**

Pretreatment with IVB in patients with PDR significantly decreased vascular endothelial cells and expressions of VEGF and HIF-1α, which further supports preoperative use of IVB in such patients.

## Introduction

Diabetic retinopathy (DR) is the leading cause of vision loss in the working-age population worldwide [1]. There is a reported 37% prevalence of DR among patients with diabetes and 54% after 10–19 years with diabetes [2]. Proliferative diabetic retinopathy (PDR) indicates occurrence of retinal neovascularization (RNV) and subsequent serum leakage, hemorrhage, and fibrovascular proliferation in the vitreous–retinal interface, which further results in macular edema, vitreous hemorrhage, and traction retinal detachment. Severe vision is inevitable when RNV occurs.

Vascular endothelial growth factor (VEGF) is an essential growth factor for angiogenesis and is particularly regulated by hypoxia inducible factor-1α (HIF-1α) under hypoxic conditions [3,4]. Increased levels of VEGF and HIF-1α were detected in the vitreous humor and in fibrovascular tissues from eyes with PDR [5–10]. VEGF and HIF-1α were indicated to provide targets for therapeutic intervention on RNV [11].

Pegaptanib and ranibizumab, two medicines for anti-VEGF, have been in use for the treatment of neovascular age-related macular degeneration since 2006 [12]. Bevacizumab (Avastin) is a human-derived full-length monoclonal antibody binding all isoforms of VEGF [13]; it is approved for use in the United States as an anti-angiogenic agent for metastatic colorectal cancer [14]. However, intravitreal injection of bevacizumab (IVB) as an off-label application has also been successfully used for the treatment of various types of ocular neovascularization, including RNV, choroidal neovascularization, and neovascular glaucoma. Several independent lines of studies have corroborated encouraging findings in IVB therapy for DR with macular edema and RNV (including perioperative use) [15].

Although the overwhelming evidence in clinical trials supports further investigation of IVB for PDR, there is little information on histopathology, especially of the effects of IVB on neovascular membranes (NVMs) of PDR. This study aimed to examine the effects of IVB on retinal vascular endothelial cells and expressions of VEGF and HIF-1α via immunostaining of NVMs surgically removed from eyes of patients with PDR.

## Methods

### Patients and materials

Patients with PDR were recruited from August 2008 to January 2009 in our department (Department of Ophthalmology, Xijing Hospital, Xi’an, Shaanxi, China). The patients all had active NVMs and associated complications, including vitreous hemorrhage, partial traction retinal detachment, and macular edema with visual acuity less than 0.1 (Table 1). No patient had iris neovascularization. All patients were indicated for vitrectomy treatment. Patients with cerebrovascular accident, myocardial infarction, deep venous thrombosis, and uncontrolled arterial hypertension and other eyes diseases were excluded. The study protocol was reviewed and approved by the Fourth Military Medical University Medical Ethics Committee (Xijing Hospital Medical Ethics No. 402). Written informed consent was obtained from all eligible patients in compliance with the Declaration of Helsinki for research involving human subjects. In total, 24 patients (24 eyes) were enrolled. None of the patients had received pan retinal photocoagulation (RPR), but seven patients (No. 4, 8, and 10 in the IVB pretreated group and No. 2, 9, 23, and 24 in the sham group) had received one session of scant retinal photocoagulation with laser spots less than 500 before admission. Patients were randomized in two groups of 12 each. One group was the IVB pretreated group with intravitreal injection of 1.25 mg bevacizumab (Avastin; Genentech, Inc., South San Francisco, CA), while the second group was the sham group with a sham injection 6 days before surgery. Ten nondiabetic patients (10 eyes) with rhegmatogenous retinal detachment complicated with proliferative vitreoretinopathy (PVR) received vitrectomy, and the epiretinal membranes were collected during vitrectomy and set as additional controls.

**Table 1:** Demography data of 24 patients with proliferative diabetic retinopathy.

**No.**	**Gender (F/M)**	**Age** **(Y)**	**Duration of DR (Mon)**	**Duration of diabete (Y)**	**Vision (Snellen)**	**Vision (LogMAR)**	**Laser**	**Medicine**	**Bevacizumab injection**
01	F	56	12	20	0.01	2.0	no	insulin	No
02	F	62	12	23	LP	2.7	Yes	Insulin	No
03	M	55	2	14	HM/30 cm	2.4	no	hypoglycemic	No
04	F	43	6	13	0.02	1.7	Yes	Insulin	Yes
05	F	40	6	10	HM/10 cm	2.4	no	insulin	Yes
06	M	49	6	15	HM/10 cm	2.4	no	Insulin	No
07	F	61	24	20	CF/15 cm	2.1	no	hypoglycemic	Yes
08	F	58	12	>1	0.02	1.7	Yes	hypoglycemic	Yes
09	M	54	24	16	HM/15 cm	2.4	Yes	hypoglycemic	No
10	M	42	1	1	0.06	1.2	Yes	hypoglycemic	Yes
11	F	46	24	>10	CF/10 cm	2.1	no	insulin	No
12	F	54	48	>15	HM/10 cm	2.4	no	hypoglycemic	No
13	M	50	14	15	HM/20 cm	2.4	no	hypoglycemic	Yes
14	F	61	21	16	CF/15 cm	2.1	no	Insulin	Yes
15	M	46	5	11	0.05	1.3	no	hypoglycemic	Yes
16	M	53	15	22	0.06	1.2	no	Insulin	No
17	F	44	24	19	CF/10 cm	2.1	no	Insulin	Yes
18	F	47	18	20	HM/15 cm	2.4	no	Insulin	No
19	M	56	9	10	0.05	1.3	no	hypoglycemic	Yes
20	F	62	12	14	0.02	1.7	no	hypoglycemic	No
21	M	55	6	12	HM/10 cm	2.4	no	hypoglycemic	Yes
22	M	48	10	15	HM/20 cm	2.4	no	Insulin	Yes
23	F	51	24	20	LP	2.7	Yes	Insulin	No
24	M	50	>24	>17	0.03	1.5	Yes	insulin	No

Gender, age, duration of diabetic retinopathy, duration of diabetes, vision and treatment of all the patients that enrolled in the study. Vision, Best-corrected visual acuity; CF, counting fingers; HM, hand motion; LP, light perception; laser, one session of scant retinal photocoagulation with less 500 spots; hypoglycemic, oral hypoglycemic drugs for control of blood glucose level.

### Specimens

The NVMs were surgically removed from each eye during pars plana vitrectomy and preserved in 4% polyoxymethylene solution for 24 h. Following dehydration in graded ethanol, cleared in xylene, and embedded in paraffin, each specimen was serially cut into 3-μm thick slices. Slides were deparaffinized, rehydrated through a graded series of ethanol, and rinsed in distilled water. Hematoxylin and eosin staining and von Willebrand (BioLegend Inc., San Diego, CA) staining were employed for vascular endothelial cell counting in NVM sections.

### Vascular endothelial cell counting**:**

For each NVM specimen, 20 consecutive slides were examined with an inverted phase-contrast microscope (Carl Zeiss AG. Oberkochen, Germany). Five visual fields (N1–N5) of each slide were chosen for analysis. Images were captured at a magnification of 200×. Results were recorded as mean ± standard deviation (SD).

### Immunohistochemistry

Immunohistochemical staining for detection of VEGF and HIF-1α expressions was done using a three-step immunoperoxidase method (labeled streptavidin biotin) as follows. Twenty consecutive slides of each specimen were incubated with 3% hydrogen peroxide to quench endogenous peroxidase. Microwave antigen retrieval was performed in citrate buffer (0.01 mol/l; pH=6.0) for 1.5 min. The sections were blocked with normal goat serum (Vector Laboratories Inc. Burlingame, CA) for 30 min at room temperature and then incubated with the primary antibody at 4 °C in humid chambers overnight. For determining expressions of VEGF and HIF-1α in specimens, rabbit polyclonal anti-VEGF antibody (1:100; Abcam Plc., Cambridge, UK) and mouse monoclonal anti-HIF-1α antibody (1:100; Abcam) were used as the primary antibody, respectively. After rinsing in PBS (0.01 mol/l, pH=7.2, NaCl 8.0 g, KCl 0.2 g, Na_2_HPO_4_ 1.44 g, KH_2_PO_4_ 0.24 g, solved in 1,000 ml distilled water) and incubating with a biotinylated secondary antibody (1:2,000; Vector Laboratories) for 30 min at 37 °C, the sections were then treated with streptavidin horseradish peroxidase (Vector Laboratories) for 30 min at 37 °C. Antigens were visualized with a diaminobenzidine kit (Sigma, St. Louis, MO). All sections were observed in a bright-field microscope (Olympus, Tokyo, Japan) and analyzed with ImagePro-Plus (Media Cybernetics, Bethesda, MD) software [16,17]. The results of average optical density (AOD) were recorded as mean±SD.

Negative controls were performed with normal rabbit serum diluted to the same concentration as the primary antibody, PBS in substitution of the primary antibody was used as blank control.

### Statistics analysis

The Student *t* test, nonparametric test (Mann–Whitney test), and stepwise regression analysis were applied to determine the difference between the two groups. A level of significance was set at p<0.05.

## Results

In the present study, we hypothesized that pretreatment with intravitreal injection of bevacizumab in PDR patients before vitrectomy can significantly decrease the amount of vascular endothelial cells and expressions of VEGF and HIF-1α in the neovascular membranes.

### Patient demography data

The patient demography data are summarized in Table 1. All eyes with PDR were classified as stage 5 according to the International Clinical Diabetic Retinopathy Disease Severity Scale [18]. Thirteen of 24 (54.1%) patients had a history of insulin therapy. The others (45.9%) took oral hypoglycemic drugs for control of blood glucose level. The best corrected visual acuity before surgery ranged from light perception to 0.06. No significant difference was found in the gender (p=0.69), age (p=0.28), preoperative vision (p=0.21), and duration of DR (p=0.11) between IVB pretreated and sham groups. The history of diabetes of the IVB pretreated group (11.92±6.02 years; range, 1–20 years) was significantly shorter than that of the sham group (17.17±3.86 years; range, 10–23 years; p=0.02; Table 2).

**Table 2:** The clinical character of the intravitreal bevacizumab (IVB) pretreated group and sham treat group were comparable.

**Clinical character**	**IVB treated (n=12)**	**sham (n=12)**	**p value**
Male (%) ‡	50.00%	41.67%	0.69
Age (Year)†	50.33±7.60	53.25±5.14	0.28
Duration of DR (Month)†	11.50±7.75	18.41±11.90	0.11
Duration of Diabetes (Year)†	11.92±6.02	17.17±3.86	0.02*
Vision (LogMAR) ‡	1.93±0.47	2.16±0.47	0.21

Gender, age, duration of diabetic retinopathy, and vision distribution of patients in the two groups had no significant difference. Duration of diabetes in the sham group was significantly longer (* p<0.05) than that of the IVB treat group. IVB=intravitreal becacizumab. All data are represented by mean±SD, except for those representing percent (%), † Student’s *t*-test, ‡: NonParametric test (Mann–Whitney test).

### Vascular endothelial cell counting

**Figure 1:**
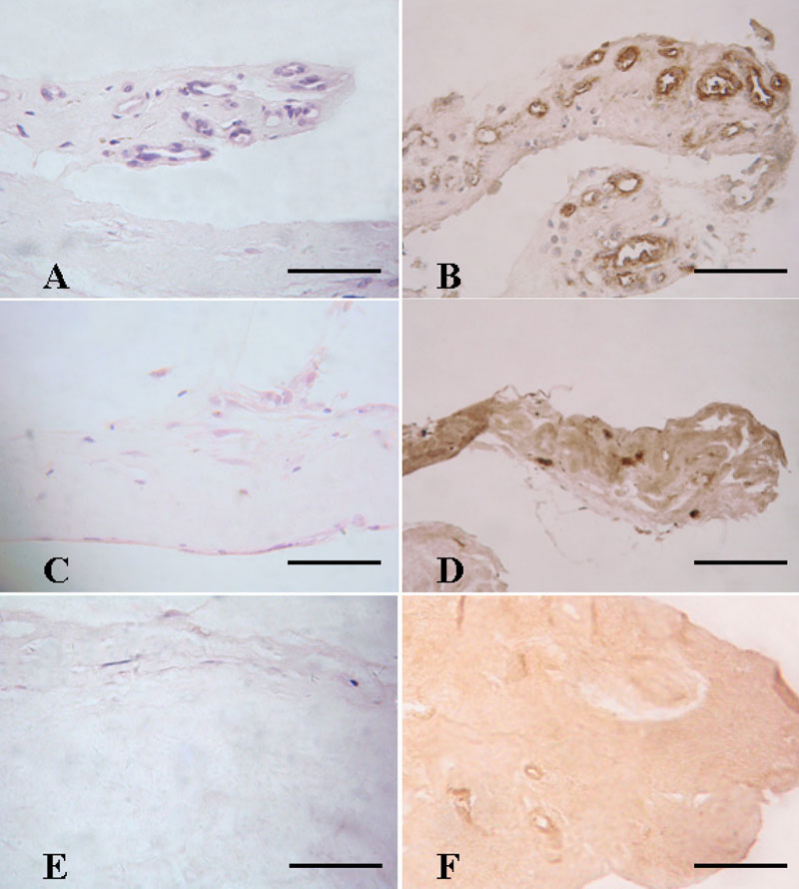
Pretreatment with intravitreal bevacizumab (IVB) significantly reduced the numbers of vascular endothelial cells in neovascular membranes (NVMs) of the eyes with proliferative diabetic retinopathy (PDR). Hematoxylin and eosin stain (H&E; **A**, **C**, and **E**) and von Willebrand stain (**B**, **D**, and **F**) were applied to detect the vascular endothelial cells in the NVMs of PDR eyes (**A**-**D**) and epiretinal membranes of the eyes with proliferative vitreoretinopathy (PVR; **E**, **F**). The untreated group (**A**, **B**) shows a significantly more number of vascular endothelial cells when compared to the IVB pretreated group (**C**, **D**; p=0.003). The epiretinal membranes of PVR eyes were set as the control group. von Willebrand stain for vascular endothelial cells in the control group was negative (**F**). Figures were selected as representative data from three independent experiments. Scale bars: 200 μm.

**Figure 2:**
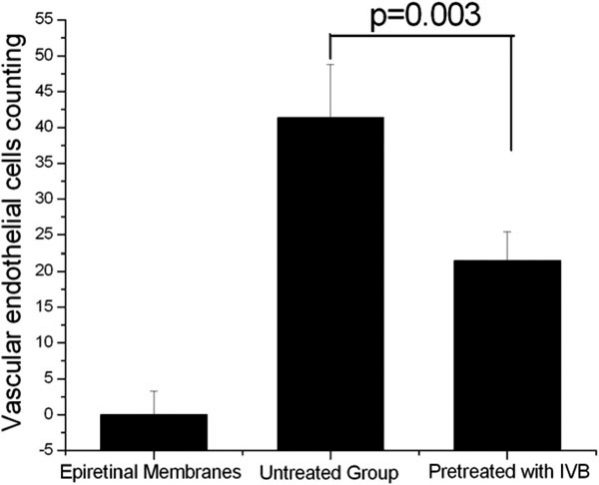
Increased vascular endothelial cell count in eyes with proliferative diabetic retinopathy (PDR) was suppressed by pretreatment of intravitreal bevacizumab (IVB). The epiretinal membranes of proliferative vitreoretinopathy (PVR) eyes were set as the control group. Quantitative analysis for the vascular endothelial cell count in the neovascular membranes (NVMs) of PDR eyes and epiretinal membranes of PVR eyes was determined by counting positively stained cells in 10 representative fields. Each value represents means±SEM from three independent experiments (p=0.003).

Compared with the IVB pretreated group, more endothelial cells were found in the sham group (21.50±3.94 versus 41.33±7.44, p=0.003; Figure 1 and Figure 2). No endothelial cells were found in epiretinal membranes of the PVR control group. A stepwise regression analysis showed that IVB was a significant negative predictor for the number of vascular endothelial cells (β=–0.89, p<0.001).

### Expressions of vascular endothelial growth factor (VEGF) and hypoxia inducible factor-1alpha (HIF-1α)

**Figure 3:**
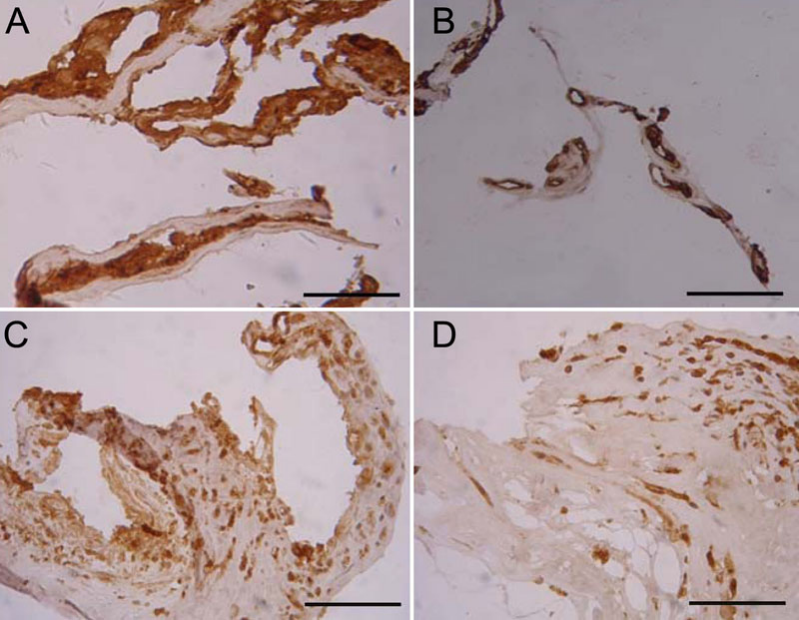
Pretreatment of intravitreal bevacizumab (IVB) remarkably reduced the levels of hypoxia inducible factor-1 alpha (HIF-1α) and vascular endothelial growth factor (VEGF) in eyes with proliferative diabetic retinopathy (PDR). Immunohistochemistry for HIF-1α (**A**, **C**) and VEGF (**B**, **D**) was performed in the neovascular membranes (NVMs) of no IVB pretreated sham group (**A**, **B**) and IVB pretreated group (**C**, **D**). The stain of both HIF-1α and VEGF in the IVB pretreated group (**C**, **D**) was significantly lower than that of the no IVB pretreated sham group (**A**, **B**). Figures were selected as representative data from three independent experiments. Scale bars: 200 μm.

**Figure 4:**
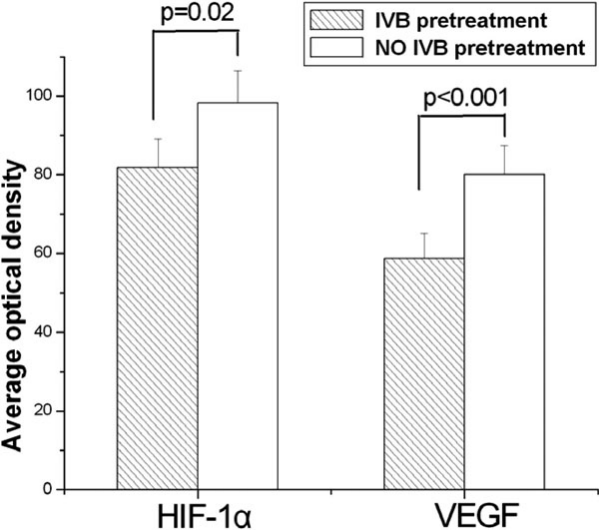
Pretreatment of intravitreal bevacizumab (IVB) remarkably reduced the expressions of hypoxia inducible factor-1 alpha (HIF-1α) and vascular endothelial growth factor (VEGF) in eyes with proliferative diabetic retinopathy (PDR). Quantitative analysis for the levels of HIF-1α and VEGF in the PDR eyes was measured by measuring the Average Optic Density values of each image using ImagePro-Plus (Media Cybernetics) software. The expression levels of both HIF-1α (p=0.02) and VEGF (p<0.001) in the IVB pretreated group was significantly lower than those of the no IVB treated group. Each value represents means±SEM from three independent experiments (n=12).

Positive staining of VEGF and HIF-1α were found in the cytoplasm of the endothelial cells in all NVM sections. Immunoreactivity intensity of HIF-1α and VEGF in the NVM sections in the IVB pretreated group were significantly lower than that of the sham group (*P*_HIF-1α_=0.02, *P*_VEGF_ < 0.001). The AOD values of VEGF and HIF-1α proteins of the two groups were 58.66±3.21 versus 80.08±3.01 and 82.25±14.18 versus 97.91±16.94, respectively. Epiretinal membranes of the PVR control group showed negative staining (Figure 3 and Figure 4). A stepwise regression analysis indicated that IVB was a significant negative predictor for the expressions of VEGF (β=–0.85, p<0.001) and HIF-1α (β=–0.64, p=0.001).

## Discussion

DR was preliminarily treated by pan retinal photocoagulation (PRP) in 1970s [19], and it was reported to reduce the risk of severe vision loss by 50% [20,21]. VEGF plays a central role in ocular neovascularization [22]. One mechanism of panretinal photocoagulation on the regression of NVM is the inhibition of VEGF production [23]. However, breakup does not occur until clinical application of anti-VEGF agents, including pegaptanib, bevacizumab, and ranibizumab. In the recent years, intravitreal injection of anti-VEGF agents has emerged as the most advanced treatment of the devastating diabetic complications. These drugs directly neutralize the function of VEGF. Current studies have shown that patients can benefit more from anti-VEGF therapy than panretinal photocogulation, such as less intervention by opacified media and concurrent macular edema. In addition, preoperative IVB injections in PDR may lessen the bleeding during an operation and decrease the occurrence of complications and repeated surgery, which improves the anatomic and functional recovery [24]. Avery et al. [25] reported a dramatic and rapid involution of retinal (73%) and iris (82%) neovascularization secondary to PDR after a single intravitreal injection of bevacizumab. Spaide et al. [26] showed that there was rapid resolution of vitreous hemorrhage and RNV regression in two patients with PDR after intravitreal bevacizumab within 1 month. Arevalo et al. [27] reported that, after treatment with IVB for patients with PDR, 61.4% patients showed total regression of RNV, 34.1% patients showed partial regression of RNV, and ETDRS best corrected visual acuity testing and optical coherence tomography demonstrated significant improvement after an average of 28.4 weeks follow up (range, 24–40 weeks). Erdol et al. [28] reported that, after a single dose of bevacizumab, complete resolution of neovascularization was 78.8% at 1 month, 63.6% at 3 months, and 45.4% at 6 months.

However, the molecular and pathological mechanism of IVB on PDR is unclear. In this study we observed that pretreatment with IVB predominantly decreased amounts of vascular endothelial cells in NVM, which is consistent with previous clinical trials [25–28]. Meanwhile, we found that the IVB-pretreated group also showed significantly lower levels of VEGF and HIF-1α in NVMs than the non-IVB-pretreated group. Our results are compatible with a recent study that the numbers of VEGF-positive vascular endothelial cells significantly decreased in the fibrovascular membranes of patients with PDR after intravitreal injection of bevacizumab [29]. HIF-1 and VEGF have been suggested to be involved [4] in angiogenesis of PDR membranes since HIF-1 mediates the angiogenic response to hypoxia by upregulating the expression of multiple angiogenic cytokines and VEGF directly promotes vascular endothelial cell proliferation, migration, and tube formation. Abu El-Asrar et al. [8] demonstrated that significantly increased intensity of HIF-1 immunoreactivity is concomitant with higher angiogenic activity in epiretinal membranes. Our results suggested that IVB pretreatment decrease the vascular endothelial cell number through down regulation of the expressions of HIF-1 and VEGF in PDR eyes.

Kohno et al. [30] found that IVB is effective in PDR and neovascular glaucoma, which leads to endothelial cell apoptosis with vascular regression. Studies showed that VEGF can inhibit endothelial cell apoptosis through protein kinase B (Akt/PKB) signaling pathway. While in Pattwell et al. [31] study of 6 PDR membranes with IVB pretreatment, histologically detectable capillaries was found in 3 clinically avascular membranes, “ghost” vessels were found in one, and the other two were both clinically and histologically still vascularized. They suggested a vaso-constrictive rather than vaso-obliterative response occurred after IVB treatment for PDR. Our study showed that the numbers of vascular endothelial cells in the NVM of PDR patients pretreated with IVB significantly decreased but were not totally eliminated. Our results are compatible with the study of Kubota et al. [29] that vascular endothelial cells with decreased expression of VEGF are still present in the NVM of PDR after IVB.

In our study four patients with IVB pretreatment (33.33%) had transient ocular hypertension (22–26 mmHg) after surgery. No other complication was noted. The Pan-American Collaborative Retina Study Group [32] studied the safety of IVB and followed up for 12 months. Repeated intravitreal injections of either 1.25 mg or 2.50 mg of bevacizumab appeared to be safe and well tolerated during the first year [32]. Systemic adverse events were reported in 18 (1.50%) patients, including seven cases (0.59%) of an acute elevation of blood pressure, six cases of cerebrovascular accident (0.50%), five cases of myocardial infarction (0.40%), two cases of iliac artery aneurysm (0.17%), two cases of toe amputation (0.17%), and five cases of death (0.40%) [32]. Ocular complications included seven cases (0.16%) of bacterial endophthalmitis, seven cases (0.16%) of traction retinal detachments, four cases (0.09%) of uveitis, one case (0.02%) of rhegmatogenous retinal detachment, and one case (0.02%) of vitreous hemorrhage [32].

Our study compared the histology of the fibrovascular membranes in PDR with or without IVB. Although the study was not a pre and post self-control study of the same specimen, the inter-group differences were limited. There were no significant differences in gender, age, duration of DR, or best corrected visual acuity between the two groups except for the duration of diabetes. The history of diabetes of the IVB group was significantly shorter than that of the group without IVB. Some patients ignored the disease in the early stages of diabetes, for example, two patients of the IVB group did not go to the hospital until they had gradually lost vision. By then, the diabetic retinopathy had developed to stage V and they were diagnosed to have type 2 diabetes mellitus. We therefore assume that the duration of diabetes in some patients is not actually the duration of disease, which is probably one of the reasons for the differences between the two groups. Several patients (three in the IVB pretreated group and four in the sham group) received one session of scant retinal photocoagulation with less than 500 spots; however, we did not considered this to be sufficient to impact the progression of PDR. Meanwhile the severity of PDR of the two groups was consistent, including the NVM, vitreous hemorrhage, macular edema, and limited traction retinal detachment without iris neovascularization. In addition, stepwise regression analysis showed that IVB was a significant negative predictor for amounts of vascular endothelial cells and expressions of VEGF and HIF-1α.

In conclusion, our study demonstrated that pretreatment with IVB in PDR patients significantly decreases amounts of vascular endothelial cells and expressions of VEGF and HIF-1α. This yields further support for the preoperative use of IVB in such patients from a histopathological point of view. Despite our sample size being small and the results limited, our findings provide additional information for clinical trials of IVB treatment for PDR. Further investigations involving a larger group with appropriate randomization and a control group will be necessary to confirm our preliminary findings.
